# Parasite detection in the ornamental fish trade using environmental DNA

**DOI:** 10.1038/s41598-019-41517-2

**Published:** 2019-03-26

**Authors:** A. Trujillo-González, R. C. Edmunds, J. A. Becker, K. S. Hutson

**Affiliations:** 1 Centre for Sustainable Tropical Fisheries and Aquaculture, College of Science and Engineering, James Cook University, 1 James Cook Dr, Townsville, QLD 4811 Australia; 2 Centre for Tropical Water & Aquatic Ecosystem Research (TropWATER), College of Science and Engineering, James Cook University, 1 James Cook Dr, Townsville, QLD 4811 Australia; 3 School of Life and Environmental Sciences, Faculty of Science, University of Sydney, 425 Werombi road, Camden, NSW 2570 Australia; 4 Cawthron Institute, 98 Halifax Street East, Nelson, 7010 New Zealand; 5 Institute for Applied Ecology, University of Canberra, 40 Bunda Street, Canberra, ACT 2601 Australia

## Abstract

Effective border control relies on stringent biosecurity protocols to detect and prevent introductions of exotic pests and diseases. Detection of pathogens and parasites in the live ornamental fish trade using environmental DNA (eDNA) techniques has the potential to improve current biosecurity practices. We examined water samples from 11 target consignments (cyprinids susceptible to *Dactylogyrus* spp. infections) and seven non-target fish consignments (non-cyprinids, not susceptible to *Dactylogyrus* spp. infections) imported from Southeast Asia to Australia for the presence of eDNA from five *Dactylogyrus* species (Monogenea: Dactylogyridae). A four-step predictive framework was used to predict putative positive and putative negative detections from quantitative PCR assays. Both target and non-target consignments were positive for *Dactylogyrus* spp. eDNA as confirmed by Sanger sequencing. Positive detections for *Dactylogyrus* spp. eDNA in non-target fish consignments demonstrates the possibility of source water contamination, limiting the applicability of eDNA screening methods at border control. This study suggests that screening for parasite eDNA within ornamental fish consignments should be tested during pre-export quarantine periods to avoid false positive detections at border control. Lastly, the proposed predictive framework has a broad utility for minimizing false positive and false negative eDNA detections of aquatic organisms.

## Introduction

The ornamental fish trade is a known route of exotic pathogen translocations globally^1–5^. Parasites and their infected hosts have been co-introduced to non-native environments with detrimental effects on biodiversity, ecosystems, industries, and dependent local communities^6^. To minimize pathogen translocation through the ornamental fish trade, governments can establish quarantine measures based on scientific risk analyses that consider the origin and history of fish stocks, parasite life cycles, host susceptibility to infection, risk of transmission to native species, and the reliability of detection methods^3,7^. Australia for example, has stringent mandatory pre-export quarantine requirements, biosecurity protocols at border control, and post arrival mandatory quarantine requirements following strict biosecurity import risk assessments of ornamental fish imports^3,8^. Despite current biosecurity protocols^9^, recent surveys of ornamental fish species imported to Australia have shown that a high diversity of parasites were not detected during inspection at border control, highlighting the need for more detection sensitivity^10^. Considering the limitation of gross visual inspection under current biosecurity protocols it is important to explore new and complimentary methods to increase biosecurity rigor and the possible integration of molecular genetic techniques.

Environmental DNA (eDNA) refers to the DNA that is naturally shed by organisms, such as through epidermal sloughing, metabolic waste excretions or post-mortem decay, into their local environment^11^. In the case of microscopic parasites, life stages like eggs, spores, cysts, larvae, juveniles and adults can be present in the water column, in sediment, or in extracellular DNA disassociated from host organisms^12^. As such, parasite genomic (gDNA) and nucleic (nDNA) can be captured with eDNA samples^12^, extracted, and screened for target species using standard molecular genetic techniques like quantitative real-time polymerase chain reaction (qPCR)^11,13,14^. Environmental DNA could enable species-level detection and monitoring in aquatic parasitology with important benefits to human health, animal welfare, freshwater fisheries, coastal aquaculture, conservation, and ecosystem health^12^. Indeed, captured and extracted eDNA from water samples has been shown to accurately detect pathogenic trematodes infecting wild amphibians^15^ and to monitor parasites infecting farmed^16–18^ and wild fish species^19^. Environmental DNA was recently proposed to be a non-destructive and sensitive detection tool for biosecurity, and was used to determine the presence of ornamental fish species present at low densities within high risk mixed imports^20^. Screening water used to import ornamental fish consignments for the presence of parasites has the potential for biosecurity monitoring advancement; however, there are no studies to date that have specifically tested this utility of eDNA.

False positive and false negative errors are commonly encountered in qPCR analyses^21–23^. From a biosecurity perspective, misinterpreting qPCR data could lead to pathogen-free consignments being considered hazards during quarantine inspection (i.e., false positive error), or high-risk pathogens going undetected in infected consignments (i.e., false negative error). As such, preventative measures must be developed to ensure accurate interpretation of qPCR data^22^ and reduce the possibility of false positive and negative results.

The aim of this study was to determine if eDNA screening by qPCR is a plausible detection tool for biosecurity. A four-step predictive framework was designed to minimize the possibility of false positive and false negative qPCR detections for the presence or absence of five ectoparasitic monogenean flukes (*Dactylogyrus anchoratus*, *D. formosus*, *D. intermedius*, *D. vastator* and *D. ostraviensis*) previously detected by necropsies infecting ornamental cyprinid fishes (*Carassius auratus* and *Pethia conchonius*) imported from Southeast Asia to Australia^10^.

## Methods

### *Dactylogyrus* spp. eDNA collection

All water samples analysed for the presence of eDNA from *Dactylogyrus* species in this study were collected during a cross-sectional survey for the presence of nationally listed aquatic pathogens associated with at least one ornamental fish host^8^. Briefly, 37 ornamental fish consignments representing 11 farmed freshwater and seven marine wild caught fish species were imported from Southeast Asia to Australia in 2015 following Australian Biosecurity Import Conditions (BICON) and subjected to Australian quarantine protocols, which involved gross visual inspection and clearance by Australian Quarantine Services^9^. A ‘consignment’ of fish was defined as a unique fish species within a shipment of fish, identified by an invoice containing details of the numbers and species of fish, date of shipment, origin and destination, accompanied by health certification^3,9^. Following release from quarantine inspection, all consignments were transported by road to an Approved Arrangement Site (AA Site) at the University of Sydney (Camden, Australia).

Freshwater consignments arrived at the AA Site in either one large plastic bag or several medium plastic bags, containing 40 to 200 individuals depending on species and size^8^. Each plastic bag contained approximately 1–5 L of freshwater and was sealed with either rubber bands or metal clasps. All consignments were housed inside large Styrofoam boxes during transit (12–48 hours including export, delivery, inspection, and release to the importer) before water samples were collected from each consignment and preserved. Negative controls (distilled water) were collected prior to collecting triplicate 15 mL samples from each fish consignment. To minimize the risk of eDNA cross contamination, each 15 mL replicate was collected from all plastic bags holding each consignment using a new disposable 20 mL sterile glass pipette attached to an automatic pipette controller (EasyPet, Eppendorf). Water samples were dispensed directly into individual pre-labelled DNA-free 50 mL centrifuge tubes, each with 33.5 mL absolute ethanol and 1.5 mL 3 M sodium acetate for preservation and then stored at room temperature^16^. Following water sample collection, 30 fish from each consignment were randomly selected, euthanized, and examined for the presence of monogenean parasites by necropsy, as described in a separate study^10^. In brief, all 30 fish were sequentially surveyed for external parasites by an experienced parasitologist using a compound microscope to carefully examine gill samples from each fish for the presence or absence of parasites^10^. A sample size of 30 fish per consignment was selected to achieve a minimum detection prevalence of 10% with 95% confidence limits determined by using exact binomial approximation^8^. As such, samples where no parasites were detected by necropsy were considered to have an apparent prevalence of 0%, with a 95% confidence interval (CI) of 0–11.4%, assuming a perfect test^8^. Environmental DNA was extracted using cetyl trimethylammonium bromide (CTAB), which included phenol-chloroform isolation and terminal isopropanol precipiation^16^. All DNA was resuspended in 60 µL 1x Tris-EDTA (TE) buffer and stored at −20 °C until screening for *Dactylogyrus* spp. eDNA by qPCR. Animal ethics, method and sampling approval was obtained from the University of Sydney Animal Ethics Committee (approval number: 720) and all methods were performed in accordance with guidelines and regulations of the University of Sydney Animal Ethics Committee.

### Design of species-specific *Dactylogyrus* primers and assay validation

Novel species-specific oligonucleotide primers were design to detect and discriminate between five *Dactylogyrus* species (Monogenea: Capsalidae): *Dactylogyrus anchoratus* (Dujardin, 1845), *Dactylogyrus formosus* Kulwiec, 1927, *Dactylogyrus intermedius* Wegener, 1909, *Dactylogyrus ostraviensis* Řehulka, 1988, and *Dactylogyrus vastator* Nybelin, 1924. All five *Dactylogyrus* spp. are highly specific to cyprinid fish hosts^24,25^. All qPCR assays targeted the internal transcribed spacer 1 (*ITS1*) between base pair 366 and 588. The *ITS1* is a high abundance nuclear gene known to be detectable in eDNA extracted from water samples^26^ and to provide species-level resolution for *Dactylogyrus*^10^ and other helminths given its low intraspecific yet high interspecific variability^27^. Each *Dactylogyrus*-specific primer was designed to target the *ITS1* region that contained the most mismatches (≥1) between target and all non-target *Dactylogyrus* species (Table 1). To achieve this, previously accessioned *Dactylogyrus* spp. *ITS1* nucleotide sequences^10^ were downloaded from GenBank (NCBI) and aligned using ClustalW (www.genome.jp/tools/clustalw, version 1.81).

**Table 1:** Primers for *Dactylogyrus* spp. *ITS1* eDNA assay.

Parasite species	Primer	Amplicon (bp)	Annealing (°C)	Primer sequence (5′–3′)	qPCR efficiency (%)	R^2^	Limit of detection (ng/µL)
*Dactylogyrus anchoratus*	D. anchoratus F	185	60	5′-GCCATCCTTGAGGGAATATGCCCA-3′	75.12	0.981	0.00065
	D. anchoratus R			5′-GAGTTTACGTTGACCGCCCGACAT-3′			
*Dactylogyrus formosus*	D. formosus F	184	65	5′-ATCATCCTTGTGGGAATCTGCCCG-3′	119.55	0.984	0.0079
	D. formosus R			5′-AAGTGTACGTTGACCGCCAGCAG-3′			
*Dactylogyrus ostraviensis*	D. ostraviensis F	120	65	5′-TCGTCGTGACGACCTTGG-3′	97.3	0.98	0.00092
	D. ostraviensis R			5′-CACATACTGCAGTGACCCT-3′			
*Dactylogyrus vastator*	D. vastator F	210	60	5′-GTTGCGGAACTGAACCCTAGCCA-3′	98.99	0.95	0.00009
	D. vastator R			5′-AGACTGCACGACACGTTACCAA-3′			
*Dactylogyrus intermedius*	D. intermedius F	210	60	5′-TCAGAATCTGAACCCTATCCAATAC-3′	104.6	0.982	1.32E-07
	D. intermedius R			5′-TGCCGCACGACACGTTA-3′			

The efficiency, R^2^ and limit of detection for each quantitative PCR assay is provided. Primer cross-reactivity tests are provided in Support Information 1.

All qPCR assays were tested for specificity *in silico* using the National Center for Biotechnology Information (NCBI) Primer BLAST^28^, Amplify4 (engels.genetics.wisc.edu/amplify), and Amplifx 1.7.0 (Nicolas Jullien; CNRS, Aix-Marseille Université: crn2m.univ-mrs.fr/pub/amplifx-dist). For Amplify4 and Amplifx 1.7.0 *in silico* tests, virtual PCRs were run against *ITS1* nucleotide sequences for all five target *Dactylogyrus* species. All assays demonstrated specificity to the targeted *Dactylogyrus* species across all three *in silico* tests. Primers were synthesized (standard desalting; Sigma-Aldrich, Australia), resuspended in 1x TE at 100 µM, and stored at −20 °C. Lastly, all qPCR assays were tested for species-specificity *in vitro* using both end-point PCR and qPCR with previously extracted genomic DNA (gDNA) from each target *Dactylogyrus* species^10^. All assays demonstrated specificity to the targeted *Dactylogyrus* species across all *in vitro* tests (Table 1; Supplementary Information 1), produced 120–210 bp amplicons and performed optimally at assay-specific annealing temperatures (60 °C or 65 °C; Table 1).

Quantitative PCR assays (10 μL or 20 μL) contained 3 or 6 μL gDNA, 0.5 or 1 μL each PCR primer (400 nM), 5 or 10 μL PowerUP® SYBR GreenER qPCR Master Mix (Life Technologies, Australia) and 1 or 2 μL MilliQ® water, respectively, and were performed under the following fast cycling conditions (ramp rate = 2.70 °C/sec): UDG incubation at 50 °C for 2 min, initial denaturation at 95 °C for 2 min, 40 cycles of 95 °C denaturation for 15 sec then 60 or 65 °C primer-specific annealing for 60 sec (Table 1), and terminal dissociation curve generation (60–95 °C at 0.15 °C/sec). Previously extracted *Dactylogyrus* spp. gDNA^10^ was quantified on a NanoDrop™ spectrophotometer (Invitrogen Inc.) and then each species-specific gDNA sample was serially diluted 1:10 to generate a five-point standard curve for each target *Dactylogyrus* species (1 × 10^−2^–1 × 10^−6^ ng/µL). Species-specific gDNA standards were used as template to determine assay amplification efficiency (E; i.e., increase in amplicon per cycle)^29^ and limit of detection (LOD; i.e., lowest gDNA standard detected across all technical qPCR replicates) for each corresponding species-specific qPCR assay. All qPCR assays were run on a QuantStudio3™ Real-Time PCR System (ThermoFisher Scientific Inc., Brisbane), and threshold cycle value (C_t_) based on a common fluorescence threshold of 0.2. Melting temperature (T_m_) values were determined for each amplicon using QuantStudio™ Design and Analysis Software (version 1.4.2). All data was exported to Microsoft Excel for comparative analyses.

### Stepwise criteria for eDNA detection and samples tested for *Dactylogyrus* spp

**Figure 1:**
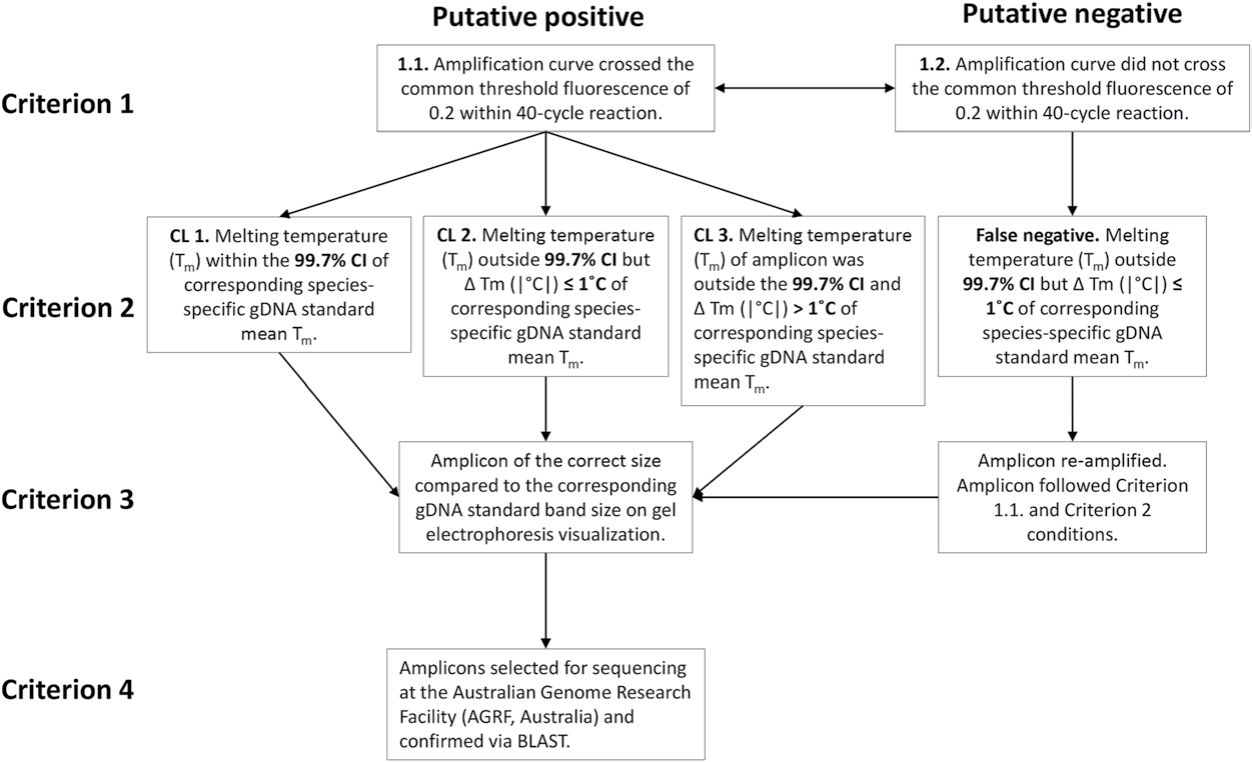
Predictive framework designed to interpret qPCR amplicon data for eDNA detection determination.

A four-step conservative predictive framework was developed to minimise the risk false positive and false negative results in qPCR T_m_ analysis^21,22,30^. These criteria were selected considering the need to accurately determine absence from disease in biosecurity^31^ and future applications of T_m_ analysis to ensure accurate and reliable detection. For each qPCR assay the T_m_ of each amplicon was compared to the mean T_m_ of the corresponding species-specific gDNA, which was calculated from all technical qPCR replicates across the entire standard curve ± 99.7% CI^32^. The absolute difference between the mean T_m_ of the species-specific gDNA standard curve and each individual qPCR technical replicate amplicon within a corresponding species-specific assay (|∆T_m_|) was calculated by subtracting the T_m_ of each technical replicate amplicon from the mean T_m_ of the corresponding species-specific gDNA standard. Calculated |∆T_m_| values were then used to categorise each putative positive detection (i.e., amplicon) into one of three confidence levels: CL 1 = high (amplicon expected to be positive for *Dactylogyrus* spp. detection), CL 2 = medium (amplicon suspected to be positive for *Dactylogyrus* spp. detection), and CL 3 = low (amplicon predicted to not be positive for *Dactylogyrus* spp. detection, i.e., false positive) (Fig. 1).

**Figure 2:**
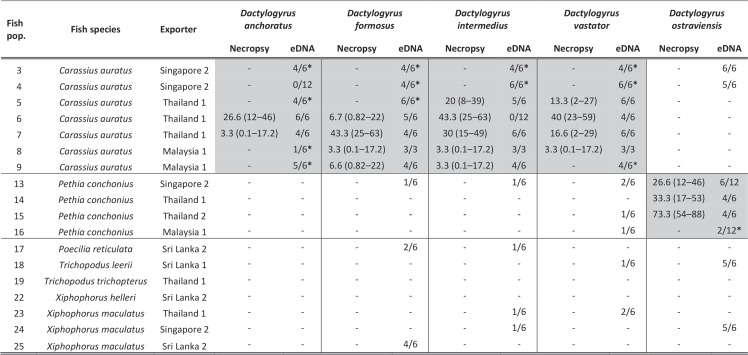
Comparison between necropsies and environmental DNA (eDNA) detection of *Dactylogyrus* species in imported ornamental fish consignments. Detections by necropsy presented as mean apparent prevalence % (95% Confidence Interval, CI)^10^ and eDNA detections as confirmed positive amplicons/total number of amplicons. Grey areas indicate qPCR assays of target fish consignments, and asterisks (*) indicate consignments where *Dactylogyrus* spp. were not detected by necropsies but were detected by eDNA assays. Negative symbols (−) indicate that no parasites were detected in a total of 30 fish and had an apparent prevalence = 0% (95% CI = 0–11.4%)^10^, and that no parasite eDNA was detected from a total of six eDNA sample replicates.

**Figure 3:**
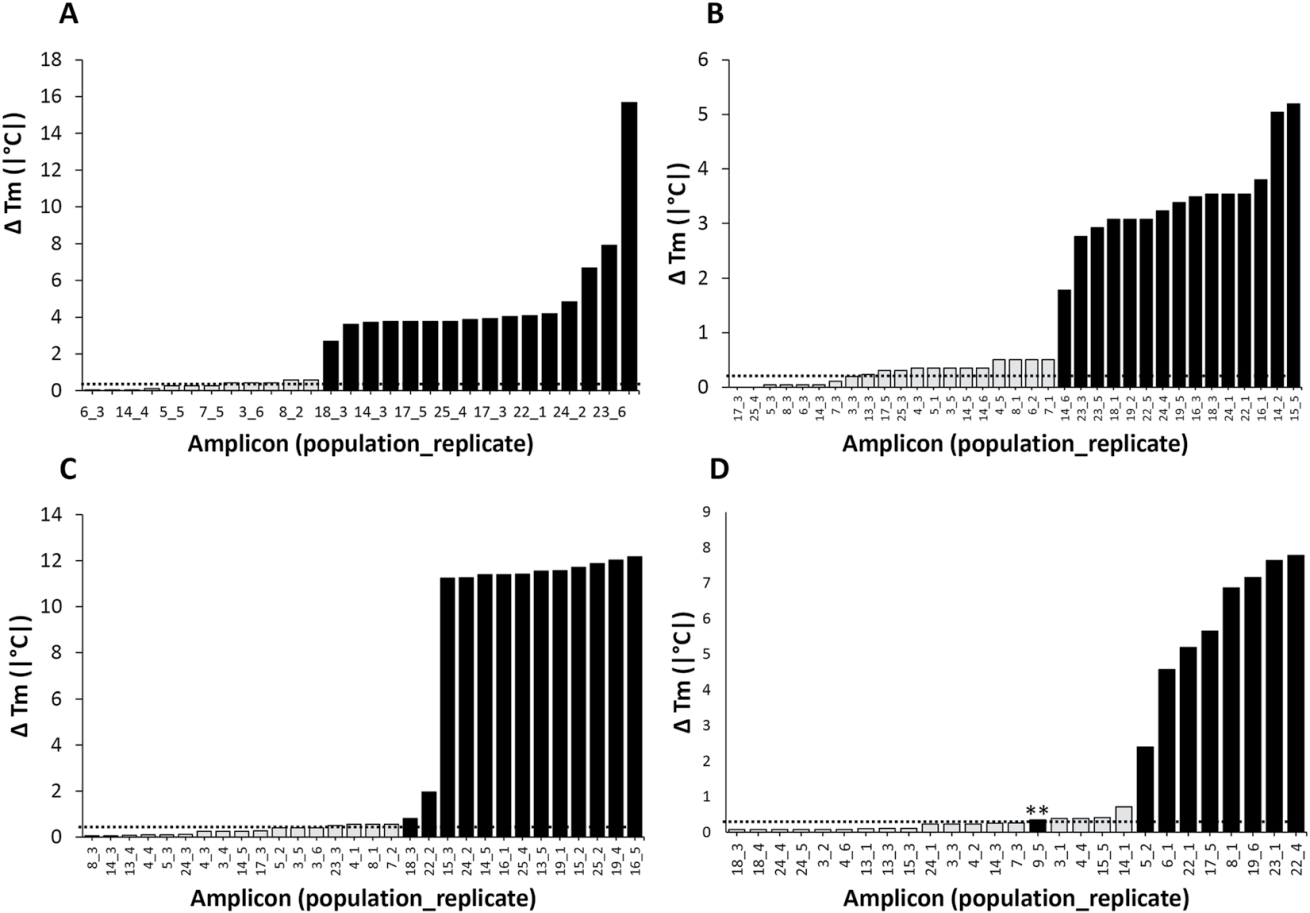
Absolute difference in melting temperature (|∆T_m_|) between sequenced amplicons derived from environmental DNA assays for *Dactylogyrus anchoratus* (**A**), *Dactylogyrus formosus* (**B**), *Dactylogyrus intermedius* (**C**) and *Dactylogyrus ostraviensis* (**D**) and their corresponding genomic DNA standards. Grey and black bars in Panels A–D represent confirmed positive and confirmed negative amplicons, respectively. Horizontal dotted lines in Panels A–D represent the upper 99.7% Confidence interval for T_m_ of species-specific standards. **Forward and reverse sequences were low in quality; however, a 72 bp fragment of consensus alignment was found to be 100% similar to *Cyprinus carpio* GenBank sequence LN599613 (i.e. considered as confirmed negative).

Amplicons were categorized as CL 1 if: (1) amplification curves crossed the common threshold fluorescence within 40-cycles (Criterion 1.1, Fig. 1), (2) T_m_ values were within 99.7% CI of the corresponding species-specific mean gDNA standard T_m_ (Criterion 2: CL 1, Fig. 1), and (3) agarose gel visualization confirmed length to match that observed and expected for corresponding species-specific gDNA standard (Criterion 3, Fig. 1). Amplicons were categorized as CL 2 if they matched CL 1 criteria (see above) but exhibited a |∆T_m_| outside 99.7% CI and ≤1 °C from mean T_m_ of corresponding species-specific standards (Criterion 2: CL 2, Fig. 1). Amplicons were categorized as CL 3 if they matched CL 1 criteria but exhibited |∆T_m_| outside 99.7% CI and >1 °C from mean T_m_ of corresponding species-specific standard (Criterion 2: CL 3, Fig. 1). Putative positive CL 1, CL 2, and CL 3 amplicons were Sanger sequenced (Australian Genome Research Facility, Brisbane) for *Dactylogyrus* spp. level confirmation (NCBI BLAST; Criterion 4, Fig. 1). If any given *Dactylogyrus* spp. eDNA assay had ≥2 putative positive amplicons categorized as CL 1 or CL 2 then two representatives for each CL were chosen for Sanger sequencing (one with lowest and one with highest |∆T_m_| value), otherwise one or both putative positive amplicons were sequenced. If any *Dactylogyrus* spp. eDNA assay had ≥2 putative positive amplicons categorized as CL 3 then the amplicons with the lowest and highest |∆T_m_| values (i.e., most and least likely to be confirmed as positive detections) were sequenced, otherwise both putative positive amplicons were sequenced.

Amplicons were considered to be putative false negative detections if no amplification curves were produced or failed to cross the common fluorescence threshold within 40 cycles (Criterion 1.2) but exhibited |∆T_m_| values within 99.7% CI of mean T_m_ of corresponding species-specific standards (false negative, Fig. 1). Amplicons categorized as putative false negatives were re-amplified by qPCR to determine if a |∆T_m_| value within 99.7% CI of mean T_m_ of corresponding species-specific standards and expected amplicon length were produced when amplified using 1 µL of PCR product from initial amplification. Putative false negative amplicons were re-amplified using six replicate 20 µL qPCRs containing 1 μL of post-PCR product, 1 μL of each PCR primer (400 nM), 10 μL PowerUP® SYBR GreenER qPCR Master Mix (Life Technologies, Australia) and 8 μL MilliQ® water, and were run under the same cycling conditions described above. Any amplicons produced from qPCR re-amplification that met Criteria 1, 2, and 3 (see above; Fig. 1) was Sanger sequenced for confirmation.

If an entire assay did not produce any amplicons that crossed common fluorescence threshold within 40 cycles (Criterion 1.2, Fig. 1) and no amplicons exhibited a discernible T_m_ then the entire assay was repeated. An assay was considered negative if neither initial or subsequent qPCR runs produced amplicons that crossed common fluorescence threshold within 40 cycles (Criterion 1.2, Fig. 1) and neither initial or subsequent qPCR runs produced amplicons with detectable T_m_ (Criterion 2, Fig. 1).

Species-specific qPCR assays were used to test extracted DNA in water samples from target and non-target fish consignments for the presence of *Dactylogyrus* spp. eDNA (Fig. 2). Imported consignments were considered ‘target’ or ‘non-target’ fish consignments based on published records of infection for any of the *Dactylogyrus* spp. targeted in this study (*n* = 5)^5,24,25,33^. Based on this criteria, seven goldfish (*Carassius auratus* (Linnaeus, 1758)) consignments were considered targets for *D. anchoratus*, *D. formosus*, *D. intermedius*, and *D. vastator* whereas four rosy barb (*Pethia conchonius* (Hamilton, 1822)) consignments were considered targets for *D. ostraviensis* (Fig. 2). Based on the same criteria, one guppy (*Poecilia reticulata*, Peters 1859), one pearl gourami (*Trichopodus leerii* (Bleeker, 1852)), one three-spot gourami (*Trichopodus trichopterus* (Pallas, 1770)), one green swordtail (*Xiphophorus hellerii* Heckel, 1848), and three platyfish (*Xiphophorus maculatus* (Günther, 1866)) consignments were considered non-target hosts for all five *Dactylogyrus* species. All target and non-target host fish consignments were screened for the presence of eDNA from all five *Dactylogyrus* species using species-specific qPCR assays (Fig. 2) followed by assessment of each produced amplicon based the selection criteria described above (Fig. 1).

## Results

### Positive *Dactylogyrus* spp. eDNA detection in target fish consignments

*Dactylogyrus* spp. eDNA was detected in all consignments where *Dactylogyrus* spp. were detected by standard necropsies. Specifically, eDNA from *D. formosus* and *D. vastator* was detected in water samples from all *C. auratus* consignments, and eDNA from *D. anchoratus* and *D. intermedius* was detected in all consignments except for consignments 4 and 6, respectively (Fig. 2). *Dactylogyrus anchoratus* was detected by both approaches (eDNA and necropsy) in consignments 6 and 7 while neither approach detected parasites in consignment 4. *Dactylogyrus ostraviensis* eDNA was detected in all target *P. conchonius* consignments, while necropsies did not detect *D. ostraviensis* in consignment 12 (Fig. 2). *Dactylogyrus* spp. eDNA was detected in five *C. auratus* and one *P. conchonius* consignments considered to have *Dactylogyrus* spp. apparent prevalence of 0% (95% CI 0–11.4%) by necropsy^10^ (Fig. 2). No eDNA was detected in negative controls.

### Positive *Dactylogyrus* spp. eDNA detections in non-target fish consignments

A total of 39 amplicons produced across all 58 qPCR tests of non-target fish consignments were confirmed positive for *Dactylogyrus* spp. eDNA (Fig. 2). *Dactylogyrus formosus*, *D. intermedius*, and *D. vastator* eDNA was detected in *P. conchonius* consignment 13 (Singapore 2; Fig. 2). *Dactylogyrus intermedius* and *D. ostraviensis* eDNA was detected in *X. maculatus* consignment 24 (Singapore 2, Fig. 2) while *D. vastator* and *D. intermedius* eDNA was detected in *X. maculatus* consignment 23 (Thailand 1; Fig. 2). Similarly, *D. ostraviensis* eDNA was detected in *C. auratus* consignments 3 and 4 as well as *X. maculatus* consignment 24 (Singapore 2; Fig. 2). Lastly, *D. formosus*, *D. intermedius*, *D. vastator*, and *D. ostraviensis* eDNA was detected by qPCR in *P. reticulata* consignment 17, *T. leeri* consignment 18, and *X. maculatus* consignment 25 (Sri Lanka; Fig. 2). No *Dactylogyrus* spp. were reported in non-target fish consignments by necropsies^10^.

### Accuracy of predictive framework

**Figure 4:**
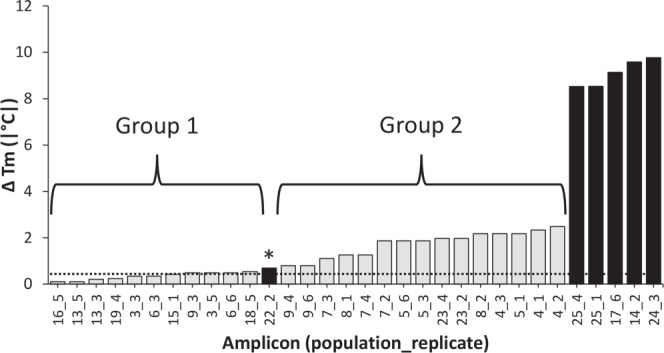
Absolute difference in melting temperature (|∆T_m_|) between *Dactylogyrus vastator* amplicons derived from environmental DNA (eDNA) assays and genomic DNA (gDNA) standards confirmed by Sanger sequencing. Grey and black bars represent confirmed positive and confirmed negative amplicons, respectively. Horizontal dotted lines represent the upper 99.7% CI for T_m_ of serially diluted *D. vastator* gDNA standard. Group 1 amplicons had 1–2 base pair differences between sequences obtained compared to *D. vastator* gDNA standard, while Group 2 amplicons had 2–18 base pair differences between sequences obtained compared to *D. vastator* gDNA. Asterisk (*): consensus sequence could not be determined for this amplicon because reverse sequence failed; however, forward sequence had 93.8% similarity to *Contraceacum* sp. [GenBank accession KM463761] and 91% similarity to *Contracaecum rudolphii* Hartwich, 1964 [GenBank accession JQ071409] and thus this amplicon was considered as a confirmed negative detection. ClustalW alignment of all *D. vastator ITS1* amplicon sequences provided in Support Information 3.

All amplicons categorized as high confidence of *Dactylogyrus* detection (CL 1) from all *Dactylogyrus* spp. qPCR assays were confirmed positive by Sanger sequencing (Fig. 1 Criterion 4). All amplicons categorized as moderate confidence (CL 2) from *D. anchoratus*, *D. formosus*, and *D. intermedius* qPCR assays were also confirmed positive by Sanger sequencing (Fig. 1 Criterion 4). Of the amplicons categorized as CL 2 from *D. ostraviensis* and *D. vastator* qPCR assays, 80% and 87.5% (*n* = 4/5 and 7/8)) were confirmed positive by Sanger sequencing, respectively. These two CL 2 amplicons were unable to be confirmed as positive detections due to poor sequencing quality (i.e., not due to non-target amplification; see Fig. 3D for *D. ostraviensis* and Fig. 4 for *D. vastator*).

No low confidence (CL 3) categorized amplicons from *D. anchoratus*, *D. formosus*, *D. intermedius*, or *D. ostraviensis* qPCR assays were confirmed positive by Sanger sequencing. However, 81.25% (*n* = 13/16) of CL 3 categorized amplicons from *D. vastator* qPCR assays were confirmed positive by Sanger sequencing (Fig. 1, Criterion 4). One *D. vastator* qPCR assay amplicon from *T. tricopterus* consignment 14 was initially considered a putative false negative (Fig. 1 Criterion 2) but was subsequently categorized as CL 1 following qPCR reamplification (Fig. 1) and confirmed positive by Sanger sequencing (Fig. 1 Criterion 4, Fig. 4 “amplicon 19_4”). All other putative false negative amplicons produced during *Dactylogyrus* spp. eDNA assays were confirmed negative following the selective framework (Fig. 1, Support Information 2).

### Amplicon sequence confirmation

All confirmed positive *D. anchoratus* amplicons were 100% homologous to *D. anchoratus ITS1* GenBank sequences (AJ564111, AJ490161, MF356241, KY859795, MF662103, MF356243, and MF356242). All confirmed positive *D. formosus* amplicons were 100% homologous to *D. formosus ITS1* GenBank sequences (AJ564135, MF356239, KM525669, KX369215, and KC876018). All confirmed positive *D. intermedius* amplicons were 100% homologous to *D. intermedius* ITS1 GenBank sequences (KC876017, KX369220, MF356236, MF356244, KJ854364, MF356237, and MF356240). All confirmed positive *D. ostraviensis* amplicons were 100% homologous to *D. ostraviensis ITS1* GenBank sequences (MF356250 and MF356249; which are the only two sequences available)^10^.

Confirmed positive *D. vastator* amplicons, unlike all other *Dactylogyrus* spp. amplicons, separated into two distinct groups (Fig. 4). *Dactylogyrus vastator* Group 1 amplicons exhibited an average T_m_ ± SD of 86.64 °C ± 0.59 with average |∆T_m_| being ± 0.6 °C away from T_m_ of gDNA standards (|∆T_m_|; Fig. 4), while amplicons in Group 2 exhibited an average T_m_ ± SD of 85.37 °C ± 0.47 with average |∆T_m_| being ± 1.97 °C away from T_m_ of gDNA standards (Fig. 4). The six confirmed positive *D. vastator* amplicons that fell within the 99.7% CI of *D. vastator* gDNA standards (Group 1) were 98–100% homologous to the following *D. vastator ITS1* GenBank sequences: MF356235 (Thailand), KY207446 (Croatia), AJ564159 (Czech Republic), MF806586 (Iran), MF356246 (Thailand), KY201104 (Italy), and KY201092 (Bosnia and Herzegovina). The 11 positive *D. vastator* amplicons that fell outside the 99.7% CI of the same *D. vastator* gDNA standards (Group 2) were 96–100% homologous to the following *D. vastator ITS1* GenBank sequences: KX369223 (China), MF356247 (Thailand), KY201103 (Czech Republic), and KM487695 (China). Groups 1 and 2 *D. vastator* amplicons differed by a total of 16 fixed nucleotide differences (Support Information 3).

## Discussion

The developed qPCR assays detected *Dactylogyrus* spp. eDNA in all consignments where necropsies detected *Dactylogyrus* spp^10^. Species-specific qPCR assays were able to detect *Dactylogyrus* spp. eDNA in six target fish consignments where necropsies considered *Dactylogyrus* spp. to have an apparent prevalence of 0% (95% CI 0–11.4; Fig. 2). As such, qPCR-based eDNA detection had higher surveillance sensitivity than necropsies, detecting *Dactylogyrus* spp. DNA in triplicate 15 mL water samples and confirming amplicons by Sanger sequencing.

However, *D. intermedius*, which was reported to infect *C. auratus* in consignment 6 by necropsy^10^ was not detected by eDNA screening in any qPCR technical replicates (*n* = 12; Fig. 2). Consequently, this was the only false negative eDNA detection observed in this study (1/90 tests; Fig. 2). It is possible that *D. intermedius* present in consignment 6 were genetically distinct from *D. intermedius* infecting consignments 5, 7, 8 and 9 (Fig. 2). The possibility of unique ITS1 genotypes in *D. intermedius* is supported by sequenced data of *D. vastator*, which displayed two *ITS1* genotypes observed across screened goldfish consignments (Fig. 4; Support Information 3). Unlike the *D. vastator* assay, the *D. intermedius* assay appears to target an *ITS1* region that is sufficiently hypervariable to prevent primer binding^27,34,35^; however, this was unknown at the time of assay development due to limited nucleotide sequence information available for *D. intermedius* populations. Such a lack of comprehensive nucleotide sequence information has also limited other molecular genetic studies aimed at investigating parasite diversity^36,37^. As such, successful implementation of the four-step predictive framework relied on the comprehensiveness of species-specific gDNA standards, suggesting |∆T_m_| analysis requires careful interpretation given the inherent dependence on sequence homology between amplicons and standards for targeted gene(s) that may or may not be known. This study highlights the need for comprehensive nucleotide sequence data and robust corresponding morphological taxonomy to ensure accuracy of designed qPCR assays and corresponding standards for |∆T_m_| analyses.

A total of 39 amplicons from non-target fish consignments were confirmed positive for *Dactylogyrus* spp. eDNA (Fig. 2). Considering that all *Dactylogyrus* spp. in this study are highly specific to cyprinid species^24,25,33^, positive detections in water samples from non-target consignments suggest that detected eDNA was not present due to active shedding from live infesting *Dactylogyrus* parasites. This interpretation is further supported by the absence of infection records for the selected *Dactylogyrus* specimens in non-target host fish species^24,25^ and non-detection by necropsies (Fig. 2). *Dactylogyrus* spp. are ectoparasites that occur naturally in southeast Asia^5,38^ and their environmental stages (i.e., eggs and oncomiracidia) could be present in recirculating aquaculture systems, raceways, or ponds used to rear freshwater species by exporting companies. As such, it is possible that exporters could have used a water source contaminated with *Dactylogyrus* spp. environmental life stages^12^ or degraded eDNA to transport exported fish consignments. If exporters do not use clean (e.g., filtered or UV treated) water to export ornamental fish consignments, then the accuracy and interpretability of eDNA assays at border control is limited, given that their applicability would depend greatly in differentiating between live infections and dead or inactive environmental parasite stages in the water column. Furthermore, considering that Australian quarantine officers have limited time to process imported consignments, eDNA-based detection by qPCR may not be applicable or reliable at border control using T_m_ analysis to carefully interpret qPCR results within an acceptable timeframe and biosecurity standard.

Screening water samples for parasite eDNA by qPCR could be a valuable detection method during pre-export quarantine periods. Current risk analyses from the Australian Government Department of Agriculture and Water Resources aim to ensure off-shore biosecurity in exporting countries^39^ by enforcing strict regulations and health requirements prior to export^9^. For example, all imported goldfish consignments must be certified free of infection from gill flukes *Dactylogyrus extensus* and *D. vastator* prior to export^9^. Both species are reported to cause significant economic losses in Asian cyprinid aquaculture^1,40^, and could pose significant risks to Australian aquarium shops selling cyprinids if live parasite infections go undetected during quarantine^1^.

Detection of eDNA by qPCR assays could be conducted on ornamental fish consignments during the mandatory quarantine period prior to export to support mandatory pre-export health certifications^9^. For instance, qPCR assays could be developed to assess the origin of parasite eDNA based on DNA decay rates by targeting various DNA fragment lengths^41–43^. Abundant long DNA fragments would indicate active shedding from live parasites while abundant short DNA fragments would indicate degrading DNA in the absence of live, shedding organisms^41,42^. Similarly, qPCR assays could also assess cellular activity by targeting environmental RNA (eRNA)^12,43,44^. Environmental RNA is indicative of active gene transcription and is proportionally less abundant in dormant stages than in metabolically active stages^12^. Given that RNA is less able to persist extracellularly and degrades quickly in dead or sloughed-off cells^12^, detection of eRNA by qPCR could be employed to determine the presence of metabolically active parasites infecting fish ready for export. Future research should consider designing qPCR assays to differentiate between active parasite infections and dead or non-active parasite stages and the applicability of eDNA detection during pre-export quarantine periods.

In conclusion, this first attempt at applying eDNA to ornamental fish parasite biosecurity highlights both the utility of incorporating molecular methods into biosecurity protocols as well as the limitations that need to be addressed if future applications and full integration are to be successful. We present a novel and comprehensive four-step predictive framework (Fig. 1) for the accurate interpretation of species-specific eDNA data and reduce false positive and false negative detections generated by Sybr-based qPCR assays. The interpretability and reliability of eDNA detection at border control specifically is limited; however, eDNA screening could prove highly valuable if implemented following pre-export quarantine periods. Further research needs to address limitations encountered in this study and test the viability of eDNA-based detection methods in other stages of quarantine and biosecurity surveillance.

## Supplementary information

Supplementary information

## Data Availability

Data for this study can be accessed as: Trujillo Gonzalez, A. (2018). Parasite detection in the ornamental fish trade using environmental DNA. James Cook University. [Data Files] 10.25903/5b90c1897397a.
