# Supplementary material for: Parasite detection in the ornamental fish trade using environmental DNA
Source: Sci Rep. 2019 Mar 26;9:5173. doi: 10.1038/s41598-019-41517-2 (PMC6435732; doi:10.1038/s41598-019-41517-2)
Supplement: Supplementary file 1 — Supplementary information [file 41598_2019_41517_MOESM1_ESM.pdf]

Parasite detection in the ornamental fish trade using environmental DNA

Trujillo-González, A.[1]*, Edmunds, R.C.[2], Becker, J.A.[3], Hutson, K.S.[1]

1. Centre for Sustainable Tropical Fisheries and Aquaculture, College of Science and Engineering, James Cook University, 1 James Cook Dr, Townsville, QLD 4811, Australia.

2. Centre for Tropical Water & Aquatic Ecosystem Research (TropWATER), College of Science and Engineering, James Cook University, 1 James Cook Dr, Townsville, QLD 4811, Australia.

3. School of Life and Environmental Sciences, Faculty of Science, University of Sydney, 425 Werombi road, Camden, NSW 2570, Australia.

***Corresponding author**:

alejandro.trujillogonzalez@my.jcu.edu.au

Phone: +61 (07) 478 15585

This file includes:

Support Information 1. Primer cross-reactivity tests. Quantitative PCR tests were run at 60 and 65 °C to test cross-reactivity for all primers used in this study, pages 1-3.

Support Information 2. Band size comparison of CL 3 and putative negative amplicons on an agarose gel. Blue font indicates amplicons selected for sequencing and red font indicates samples considered negative, pages 4-5.

Support Information 3. *Dactylogyrus vastator* alignment of sequenced Internal Transcribed Spacer 1 amplicons and accessioned sequences in Genbank from the National Center for Biotechnology Information, pages 6-7.

Support Information 1. Primer cross-reactivity tests. Quantitative PCR tests were run at 60 and 65 °C to test cross-reactivity for all primers used in this study. Primers were initially tested for another species of *Dactylogyrus* (*D. baueri*), but cross-reactivity was not eliminated. As such, tests for *D. baueri* were removed from this study.

Genomic DNA

1: *D. anchoratus*
2: *D. baueri*
3: *D. intermedius*
4: *D. formosus*
5: *D. ostraviensis*
6: *D. vastator*

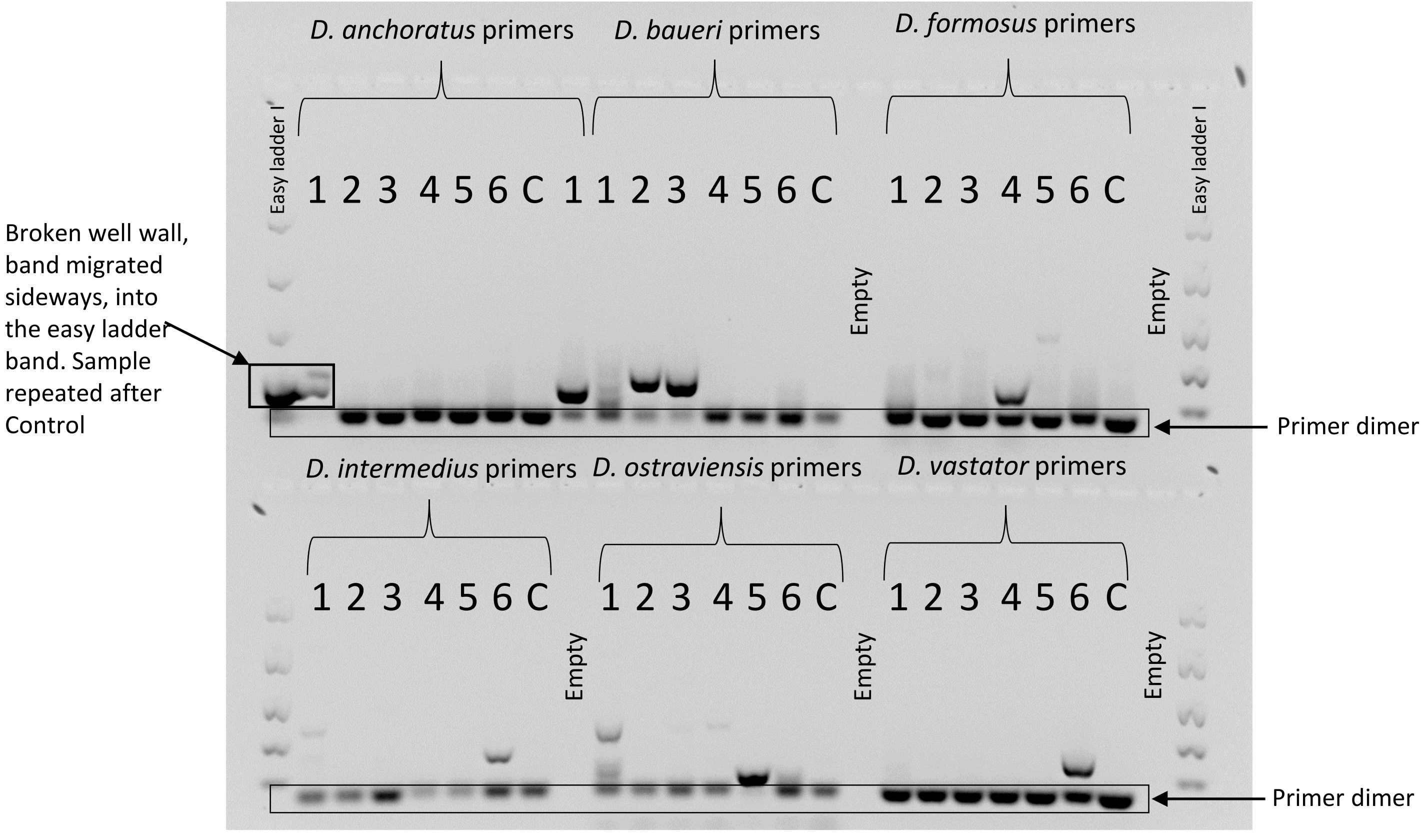

Cross-reactivity test 1

qPCR cycling conditions:
95/4min, (95/15s-60/30s-72/30s) 40 cycles, 72/7 min

Results:
D. anchoratus: Specific, test annealing at 65 °C to confirm
D. baueri: non-specific (intermedius). Develop new primer pair.
D. intermedius: Amplified wrong DNA, develop new primer.
D. formosus: Non-specific, test annealing at 65 °C.
D. ostraviensis: Non-specific, test , test annealing at 65 °C.
D. vastator: specific.

Genomic DNA

1: *D. anchoratus*
2: *D. baueri*
3: *D. intermedius*
4: *D. formosus*
5: *D. ostraviensis*
6: *D. vastator*

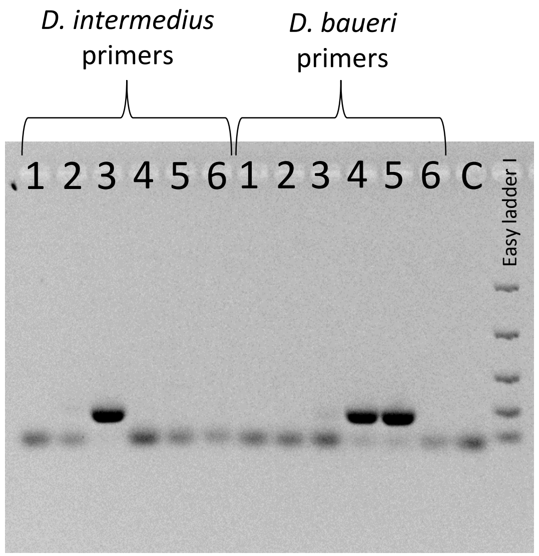

Cross-reactivity test 2

qPCR cycling conditions:
95/4min, (95/15s-60/30s-72/30s) 40 cycles, 72/7 min

Primers:
Dactylogyrus intermedius primer pair 2

Dactylogyrus baueri primer pair 2

Results:
D. baueri: non-specific, and did not amplify *D. baueri* DNA. Remove altogether.
D. intermedius: Specific at 60 °C. Use this primer pair.

Genomic DNA

1: *D. anchoratus*
2: *D. baueri*
3: *D. intermedius*
4: *D. formosus*
5: *D. ostraviensis*
6: *D. vastator*

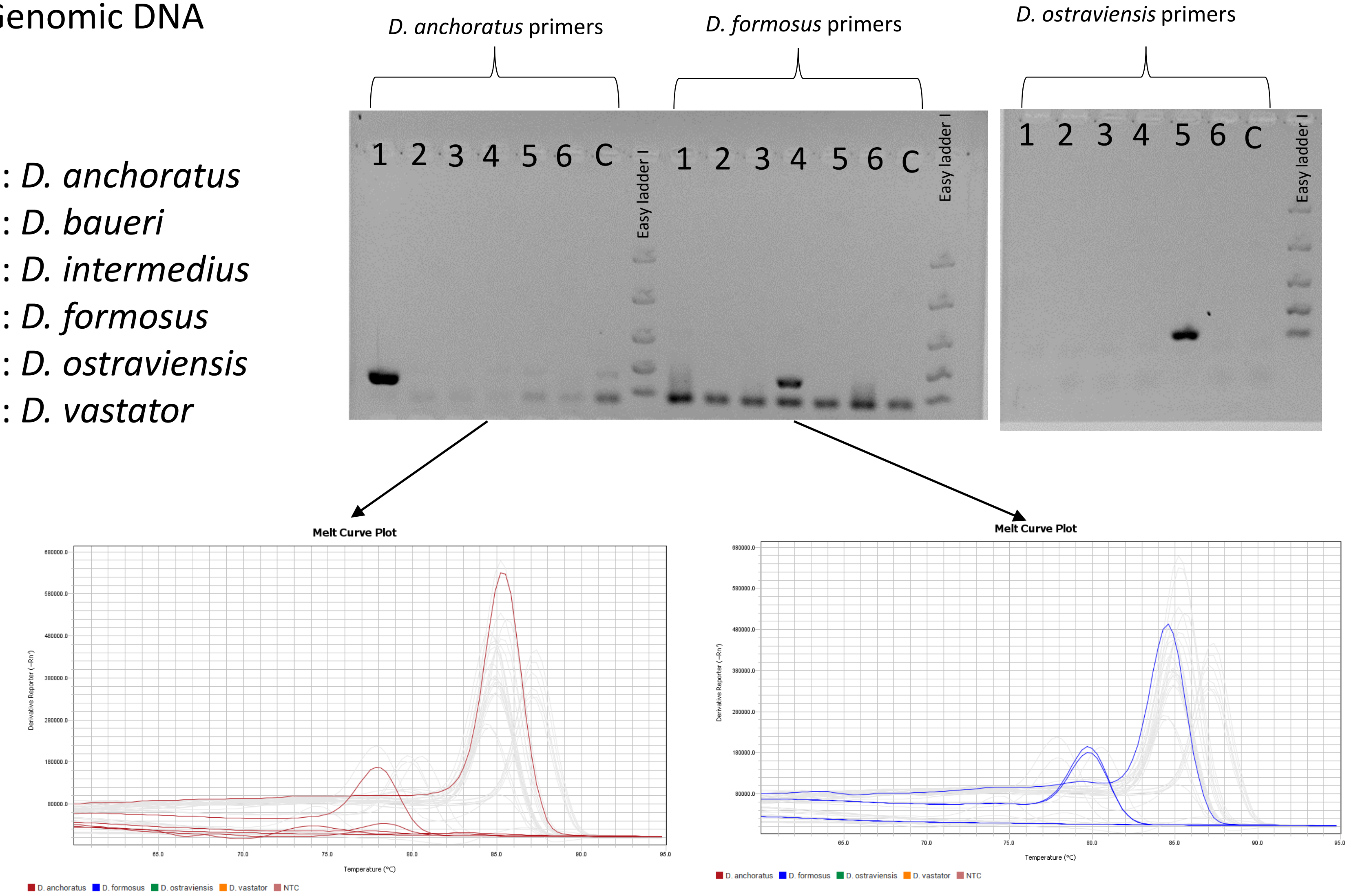

Cross-reactivity test 3

qPCR cycling conditions:
95/4min, (95/15s-65/30s-72/30s) 40 cycles, 72/7 min

Results:
D. anchoratus: Possible contamination, Tm of background bands is different from band in *D. anchoratus*. Specific at 65 °C.
D. formosus: Tm of background bands is different from band in *D. anchoratus*. Specific at 65 °C.
D. ostraviensis: Non-specific, test , test annealing at 65 °C.
D. vastator: specific.

Support Information 2. Band size comparison of CL 3 and putative negative amplicons on an agarose gel. Blue font indicates amplicons selected for sequencing and red font indicates samples considered negative.

1. D. anchoratus_24_2
2. D. anchoratus_19_5
3. D. anchoratus_22_1
4. D. anchoratus_22_5
5. D. anchoratus_14_1
6. D. anchoratus_14_3
7. D. anchoratus_16_1
8. D. anchoratus_16_4
9. D. anchoratus_std 1
10. D. formosus_18_1
11. D. formosus_18_3
12. D. formosus_24_1
13. D. formosus_24_4
14. D. formosus_19_2
15. D. formosus_19_5
16. D. formosus_23_3
17. D. formosus_23_5
18. D. formosus_22_1
19. D. formosus_22_5
20. D. formosus_14_2
21. D. formosus_14_6
22. D. formosus_15_5
23. D. formosus_16_1
24. D. formosus_16_3
25. D. formosus_std 1
26. D. intermedius_24_2
27. D. intermedius_19_1
28. D. intermedius_19_4
29. D. intermedius_25_2
30. D. intermedius_25_4
31. D. intermedius_13_5
32. D. intermedius_14_5
33. D. intermedius_15_2
34. D. intermedius_15_3
35. D. intermedius_16_1
36. D. intermedius_16_5
37. D. intermedius_std 1
38. D. vastator_4_2
39. D. vastator_4_3
40. D. vastator_5_1
41. D. vastator_5_6
42. D. vastator_8_2
43. D. vastator_7_2
44. D. vastator_7_4
45. D. vastator_9_6
46. D. vastator_23_2
47. D. vastator_23_4
48. D. vastator_25_1
49. D. vastator_25_4
50. D. vastator_std 1
51. D. ostraviensis_13 rerun_1
52. D. ostraviensis_13 rerun_3
53. D. ostraviensis_15_5
54. D. ostraviensis_17_5
55. D. ostraviensis_22_1
56. D. ostraviensis_22_4
57. D. ostraviensis_13_rerun_reamp_1
58. D. ostraviensis_13_rerun_reamp_3
59. D. ostraviensis_std 1

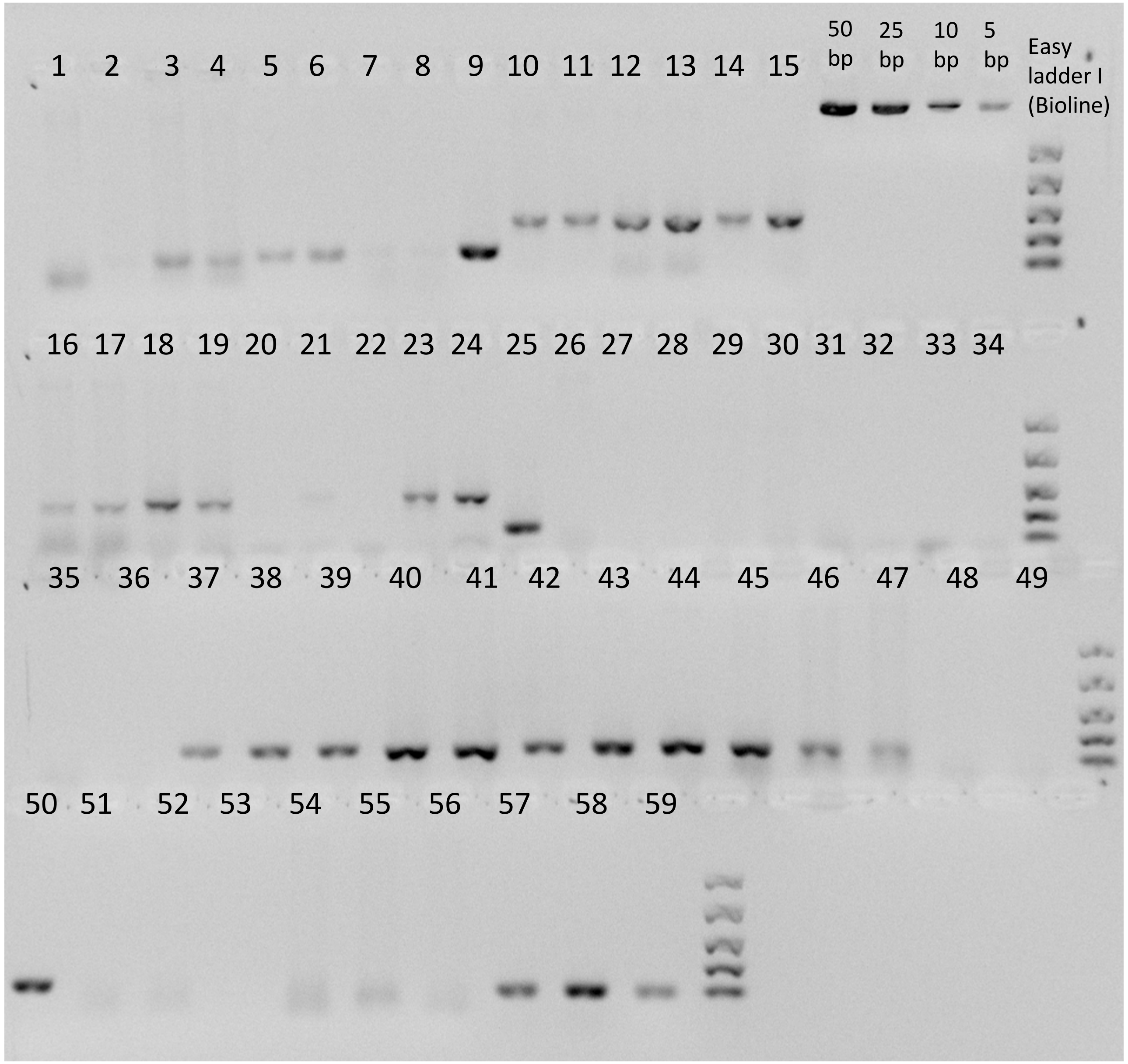

1. D. anchoratus_24_2
2. D. anchoratus_19_5
3. D. anchoratus_22_1
4. D. anchoratus_22_5
5. D. anchoratus_14_1
6. D. anchoratus_14_3
7. D. anchoratus_16_1
8. D. anchoratus_16_4
9. D. anchoratus_18_3
10. D. anchoratus_23_6
11. D. anchoratus_13_2
12. D. anchoratus_15_3
13. D. anchoratus_std 1
14. D. anchoratus_NTC

15. D. formosus_14_2
16. D. formosus_14_6
17. D. formosus_15_5
18. D. formosus_std 1
19. D. formosus_NTC

20. D. intermedius_24_2
21. D. intermedius_19_1
22. D. intermedius_19_4
23. D. intermedius_25_2
24. D. intermedius_25_4
25. D. intermedius_13_5
26. D. intermedius_14_5
27. D. intermedius_15_2
28. D. intermedius_15_3
29. D. intermedius_16_1
30. D. intermedius_16_5
31. D. intermedius_6_eDNA_1
32. D. intermedius_6_eDNA_2
33. D. intermedius_6_eDNA_3
34. D. intermedius_6_eDNA_4
35. D. intermedius_6_eDNA_5
36. D. intermedius_6_eDNA_6
37. D. intermedius_18_3
38. D. intermedius_22_2
39. D. intermedius_std 1
40. D. intermedius_NTC
41. D. vastator_23_2
42. D. vastator_23_4
43. D. vastator_25_1
44. D. vastator_25_4
45. D. vastator_14_2
46. D. vastator_8_1
47. D. vastator_std 1
48. D. vastator_NTC

49. D. ostraviensis_13_1
50. D. ostraviensis_13_3
51. D. ostraviensis_15_5
52. D. ostraviensis_17_5
53. D. ostraviensis_22_1
54. D. ostraviensis_22_4
55. D. ostraviensis_16_eDNA_1
56. D. ostraviensis_16_eDNA_2
57. D. ostraviensis_16_eDNA_3
58. D. ostraviensis_16_eDNA_4
59. D. ostraviensis_16_eDNA_5
60. D. ostraviensis_16_eDNA_6
61. D. ostraviensis_19_6
62. D. ostraviensis_23_1
63. D. ostraviensis_25_4
64. D. ostraviensis_5_2
65. D. ostraviensis_8_1
66. D. ostraviensis_6_1
67. D. ostraviensis_7_3
68. D. ostraviensis_9_5
69. D. ostraviensis_std 1
70. D. ostraviensis_NTC

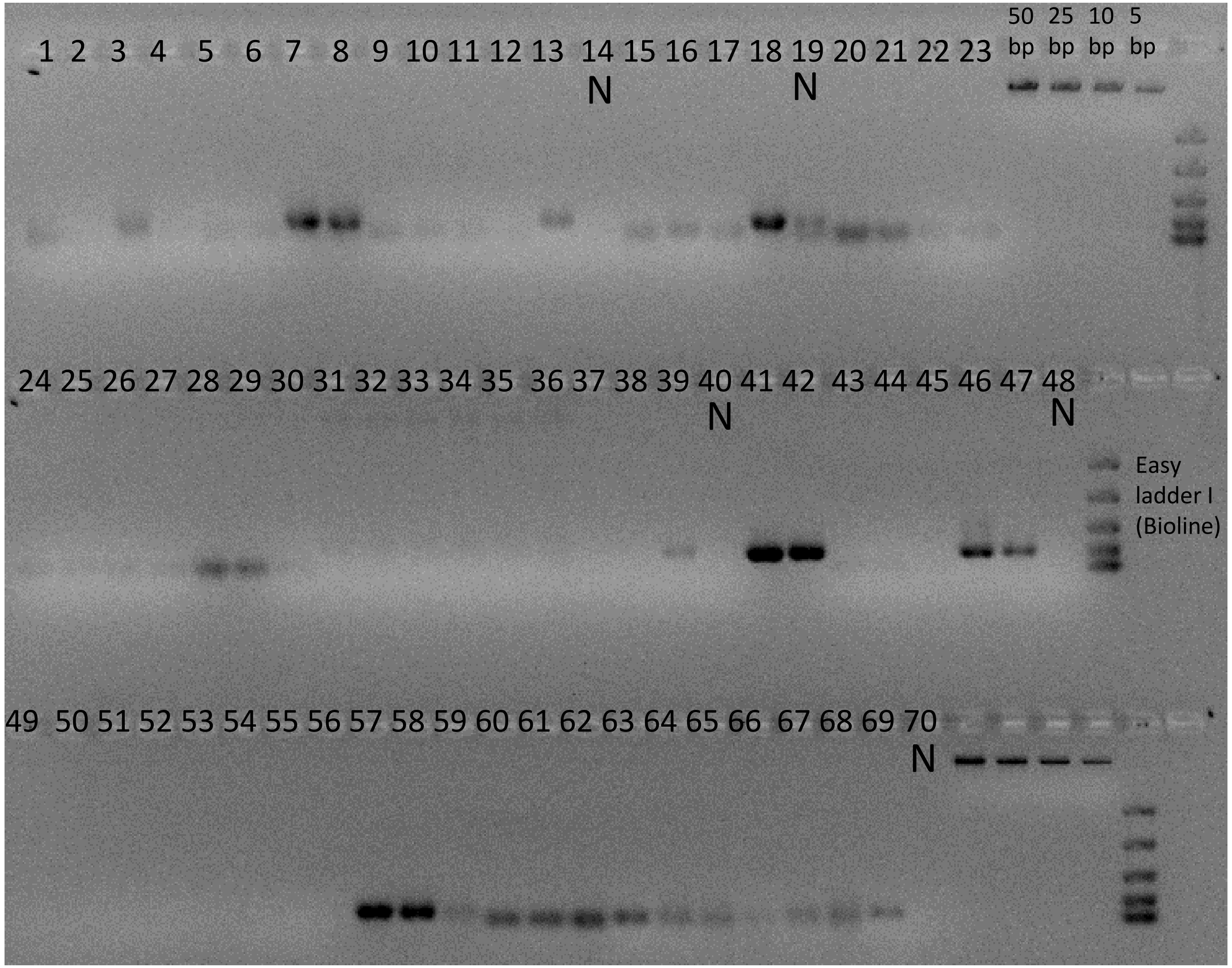

Support Information 3. *Dactylogyrus vastator* alignment of sequenced Internal Transcribed Spacer 1 amplicons and accessioned sequences in Genbank from the National Center for Biotechnology Information.

```
                                        10        20        30        40        50        60        70        80        90
                                ....|....|....|....|....|....|....|....|....|....|....|....|....|....|....|....|....|....|
D. vastator standard            ACCCTAGCCAAGGATCGTGTCAGTCGGCCTTCACCTCGGGAGGCTTACGCCCTCCAAGTTGGCCACCTATGGTACAGAAATGTACGGTGC
13_5_Singapore 2                ..........................................................................................
16_5_Malaysia 1                 ..........................................................................................
13_3_Singapore 2                ..........................................................................................
19_4_Thailand 1                 ..........................................................................................
3_3_Singapore 2                 ..........................................................................................
6_3_Thailand 1                  ...............T..........................................................................
15_1_Thailand 2                 ...............T..........................................................................
9_3_Malaysia 1                  ...............T............................----------------------------------------------
3_5_Singapore 2                 ..........................................................................................
6_6_Thailand 1                  ...............T.......................................-----------------------------------
18_5_Sri Lanka 1                ...............T.......................................-----------------------------------
MF356235_Thailand               ..........................................................................................
KY207446_Croatia                ..........................................................................................
AJ564159_Czech Republic         ..........................................................................................
MF806586_Iran                   ..........................................................................................
MF356246_Thailand               ..........................................................................................
KY201104_Italy                  ..........................................................................................
KY201092_Bosnia and Herzegovina ..........................................................................................
9_4_Malaysia 1                  ...............T.............................C....................------------------------
9_6_Malaysia 1                  ...............T.....................................................---------------------
7_3_Thailand 1                  ...............T...A.........................C....................T.....................A.
7_4_Thailand 1                  ...........T...T...A.........................C....................T.....................A.
8_1_Malaysia 1                  ...............T.............................C....................T.....................A.
5_3_Thailand 1                  ...........T...T...A.........................C....................T.....................A.
5_6_Thailand 1                  ...........T...T...A.........................C....................T.....................A.
7_2_Thailand 1                  ...............T...A.........................C....................T.....................A.
23_2_Thailand 1                 ...........T...T...A.........................C....................T.....................A.
23_4_Thailand 1                 ...........T...T...A.........................C....................T.....................A.
4_3_Singapore 2                 ...........T...T...A.........................C....................T.....................A.
5_2_Thailand 1                  ...........T...T...A.........................C....................T.....................A.
8_2_Malaysia 1                  ...........T...T...A.........................C....................T.....................A.
4_1_Singapore 2                 ...........T...T...A.........................C....................T.....................A.
4_2_Singapore 2                 ...........T...T...A.........................C....................T.....................A.
KX369223_China                  ...........T...T...A.........................C....................T...............C.....A.
MF356247_Thailand               ...........T...T...A.........................C....................T.....................A.
KY201103_Czech Republic         ...........T...T...A.........................C....................T.....................A.
KM487695_China                  ...........T...T...A.........................C....................T.....................A.
```

```
                                        100       110       120       130       140       150       160       170
                                ....|....|....|....|....|....|....|....|....|....|....|....|....|....|....|....|....|...
D. vastator standard            GGTCCTGTCAGTAACACTTCTTACCGGCAGCGGCTCGTGTCGTTCATCCGCCGACCCACTGGATCGGCCGTCTGGACTGTGCGAATTG
13_5_Singapore 2                ...........................................................................G............
16_5_Malaysia 1                 ...........................................................................G............
13_3_Singapore 2                ...........................................................................G............
19_4_Thailand 1                 ...........................................................................G............
3_3_Singapore 2                 ........................................................................................
6_3_Thailand 1                  ........................................................................................
15_1_Thailand 2                 ...........................................................................G............
9_3_Malaysia 1                  ----------------------------------------------------------------------------------------
3_5_Singapore 2                 ........................................................................................
6_6_Thailand 1                  ----------------------------------------------------------------------------------------
18_5_Sri Lanka 1                ----------------------------------------------------------------------------------------
MF356235_Thailand               ...........................................................................G............
KY207446_Croatia                ...........................................................................G............
AJ564159_Czech Republic         ...........................................................................G............
MF806586_Iran                   ........................................................................................
MF356246_Thailand               ........................................................................................
KY201104_Italy                  ........................................................................................
KY201092_Bosnia and Herzegovina ........................................................................................
9_4_Malaysia 1                  ----------------------------------------------------------------------------------------
9_6_Malaysia 1                  ----------------------------------------------------------------------------------------
7_3_Thailand 1                  .........G...............TT...........A....C.....A..................T.......A..CA...-...
7_4_Thailand 1                  .........G...............TT...........A....C.....A..................T.......A..CA...-...
8_1_Malaysia 1                  .........G...............TT...........A....C.T...A..................T...G...A..CA...-...
5_3_Thailand 1                  .........G...............TT...........A....C.....A..................T.......A..CA...-...
5_6_Thailand 1                  .........G...............TT...........A....C.....A..................T.......A..CA...-...
7_2_Thailand 1                  .........G...............TT...........A....C.....A..................T.......A..CA...-...
23_2_Thailand 1                 .........G...............TT...........A....C.....A..................T.......A..CA...-...
23_4_Thailand 1                 .........G...............TT...........A....C.....A..................T.......A..CA...-...
4_3_Singapore 2                 .........G...............TT...........A....C.....A..................T.......A..CA...-...
5_2_Thailand 1                  .........G...............TT...........A....C.....A..................T.......A..CA...-...
8_2_Malaysia 1                  .........G...............TT...........A....C.....A..................T.......A..CA...-...
4_1_Singapore 2                 .........G...............TT...........A....C.....A..................T.......A..CA...-...
4_2_Singapore 2                 .........G...............TT...........A....C.....A..................T.......A..CA...-...
KX369223_China                  .........G...............TT...........A....C.....A..................T.......A..CA...-...
MF356247_Thailand               .........G...............TT...........A....C.....A..................T.......A..CA...-...
KY201103_Czech Republic         .........G...............TT...........A....C.....A..................T.......A..CA...-...
KM487695_China                  .........G...............TT...........A....C.....A..................T.......A..CA...-...
```
